# Biochar Amendment Alters the Nutrient-Use Strategy of Moso Bamboo Under N Additions

**DOI:** 10.3389/fpls.2021.667964

**Published:** 2021-06-23

**Authors:** Jinpei Gao, Quan Li, Junbo Zhang, Kunkai Cui, Zhizhuang Wu, Man Shi, Xinzhang Song

**Affiliations:** ^1^State Key Laboratory of Subtropical Silviculture, Zhejiang A&F University, Hangzhou, China; ^2^Center for Ecological Forecasting and Global Change, College of Forestry, Northwest A&F University, Yangling, China; ^3^Key Laboratory of High Efficient Processing of Bamboo of Zhejiang Province, China National Bamboo Research Center, Hangzhou, China

**Keywords:** resorption efficiency, resorption proficiency, N deposition, biochar application, Moso bamboo, nutrient-use strategy

## Abstract

Nutrient resorption can affect plant growth, litter decomposition, and nutrient cycling. Although the effects of nitrogen (N) and biochar fertilizers on soil nutrient concentrations and plant nutrient uptake have been studied, an understanding of how combined applications of N and biochar affect plant nutrient resorption in plantations is lacking. In this study, we applied N (0, 30, 60, and 90 kg N ha^−1^ yr^−1^ defined as N0, N30, N60, and N90, respectively) and biochar (0, 20, and 40 t biochar ha^−1^ defined as BC0, BC20, and BC40, respectively) to the soil of a Moso bamboo plantation. We investigated the effects of these treatments on N and phosphorus (P) resorption by young and mature bamboo plants, as well as the relationships between nutrient resorption and leaf and soil nutrient concentrations. Young bamboo showed significantly greater foliar N resorption efficiency (NRE) and P resorption efficiency (PRE) than mature bamboo. N addition alone significantly increased the N resorption proficiency (NRP) and P resorption proficiency (PRP) but significantly decreased the NRE and PRE of both young and mature bamboo. In both the N-free and N-addition treatments, biochar amendments significantly reduced the foliar NRE and PRE of young bamboo but had the opposite effect on mature bamboo. Foliar NRE and PRE were significantly negatively correlated with fresh leaf N and P concentrations and soil total P concentration but significantly positively correlated with soil pH. Our findings suggest that N addition inhibits plant nutrient resorption and alters the nutrient-use strategy of young and mature bamboo from “conservative consumption” to “resource spending.” Furthermore, biochar amendment enhanced the negative effect of N addition on nutrient resorption in young bamboo but reduced the negative effect on that of mature bamboo under N-addition treatments. This study provides new insights into the combined effects of N and biochar on the nutrient resorption of Moso bamboo and may assist in improving fertilization strategies in Moso bamboo plantations.

## Introduction

Nutrient resorption – a physiological process by which plants reallocate nutrients from senescent structures to other living tissues for later use (Clark, 1977; Turner, 1977; Yuan and Chen, 2015) – can improve nutrient utilization (Chapin, 1980; Vitousek, 1984; Wu et al., 2020) and reduce plant nutrient uptake from the environment (Brant and Chen, 2015; Yuan and Chen, 2015; Lü et al., 2020). Furthermore, nutrient resorption is an important strategy employed by plants to overcome nutrient limitations and meet their nutritional demands (Killingbeck, 1996; Vergutz et al., 2012), and is most commonly quantified using nutrient resorption efficiency (RE) and resorption proficiency (RP; Lü et al., 2020; Wu et al., 2020). Nutrient RE is the difference between the amount of a given nutrient in green versus fully senesced tissue relative to the amount in green tissue, and nutrient RP is the absolute level of a nutrient found in senesced leaves (Killingbeck, 1996; Chang et al., 2017). Plants usually adopt a “conservative consumption” (high RE and low RP) or “resource spending” (low RE and high RP) nutrient-use strategy (Wright and Westoby, 2003).

Nitrogen (N) and phosphorus (P) resorption play an important role in plant growth (Sterner and Elser, 2002); contribute to leaf N and P contents; and influence plant photosynthesis, reproduction, and physiological processes (Koerselman and Meuleman, 1996; Kerkhoff et al., 2006; Tian et al., 2018). Resorption is estimated to supply 31 and 40% of annual plant N and P demands, respectively, on a global scale (Cleveland et al., 2013). Meanwhile, the NRE:PRE ratio is usually regarded as an indicator of N or P limitation (Du et al., 2020; Li et al., 2021). An NRE:PRE ratio >1 indicates a stronger N limitation than P limitation; P is more limiting when the NRE:PRE ratio is <1 (Du et al., 2020). Changes in nutrient (N and P) supply influence plant nutrient resorption (Chen et al., 2007). However, these findings remain highly controversial (Yuan and Chen, 2015; Lü et al., 2020), especially for N addition.

An increase in atmospheric N deposition, mainly from the burning of fossil fuels and artificial fertilizer use, is an important phenomenon in global climate change (Janssens et al., 2010). The latest research showed that the average annual N deposition in China reached 20.4 ± 2.6 kg N ha^−1^ yr^−1^ in 2011–2015, which far exceeded that of Europe and the United States; thus, N deposition rates in China are among the highest in the world (Yu et al., 2019). High N deposition can change plant N and P resorption by changing the N and P contents of plants and soils, which in turn influences N and P cycles in ecosystems (McNeil et al., 2007; Farrer et al., 2013; Yuan and Chen, 2015; Du et al., 2016; Lü et al., 2020; Zhao et al., 2020). A meta-analysis by Yuan and Chen (2015) showed that N enrichment inhibited plant N resorption; however, other studies have observed both neutral and positive effects on plant N resorptions (van Heerwaarden et al., 2003; Li et al., 2010; Lü and Han, 2010; Lü et al., 2013, 2020). N addition also promoted (Lü et al., 2013), inhibited (Sardans et al., 2016), and/or did not affect (Lü and Han, 2010; Zhang et al., 2017b) foliar P resorption in forests. However, these studies have only partially observed N or P resorption; few studies have simultaneously considered the resorption of both N and P in forests.

Biochar is produced by the pyrolysis of organic matter in a high-temperature and oxygen-limited environment (Antal and Grønli, 2003) and is widely applied in forestry ecosystems (Li et al., 2018a) for soil amendment (Jeffery et al., 2015). Biochar has a high surface area and high pH and contains various forms of N and P nutrients (e.g., NH_4_^+^ and ortho-P; Gul and Whalen, 2016). Over the past few decades, most studies have focused on the effects of biochar amendments on soil physical and chemical properties, the soil organic carbon pool, and soil greenhouse gas emissions (Song et al., 2016b; Li et al., 2018a). For example, biochar application enhanced soil fertility by increasing soil pH and cation-exchange capacity (CEC), thereby increasing soil N and P concentrations (Chan et al., 2007; Nelson et al., 2011; Biederman and Harpole, 2013), which affected foliar N and P concentrations (Major et al., 2010; Zhang et al., 2019b). However, there are relatively few studies addressing the potential effects of biochar application on plant N and P resorption. By understanding these mechanisms, we can predict potential changes in plant productivity in biochar-amended forests.

Moso bamboo (*Phyllostachys edulis*) – one of the most economically important bamboo species – is widely distributed in tropical and subtropical regions of East and Southeast Asia (Song et al., 2011, 2020). In China, it covers an area of 4.68 million hectares, accounting for 73% of the total bamboo forest area (Li and Feng, 2019). Owing to its rapid growth and strong regenerative ability (Song et al., 2016c), Moso bamboo is the main source of nontimber forest products in China (Song et al., 2015) and has a high potential for C sequestration (Song et al., 2017a). The subtropics of China, the main growing region of Moso bamboo, are subject to high N deposition; the area has an average rate of 30 kg N ha^−1^ yr^−1^ (Jia et al., 2014), which is expected to continue to increase in the next few decades (Galloway et al., 2008; Liu et al., 2013). Our previous study found that N addition increased foliar N and P concentrations and soil available N (AN) and P (AP), but decreased soil pH in Moso bamboo plantations (Li et al., 2016; Song et al., 2016a). In addition, biochar applications significantly increased soil pH and bacterial diversity and decreased soil urease and acid phosphatase activities (Li et al., 2018b; Peng et al., 2019). To mitigate soil acidification caused by N addition, biochar was applied to soil in a Moso bamboo plantation that had received N input for approximately 21 months. Our previous studies found that the combination of N addition and biochar amendment increased soil pH and affected soil nutrient and enzyme activities (Li et al., 2018b; Peng et al., 2019), which influence leaf nutrient resorption. However, the effects of N deposition and biochar amendment and their interactions on leaf nutrient resorption in Moso bamboo forests are still unclear.

In this study, we applied N and biochar to a Moso bamboo plantation to investigate leaf nutrient resorption by Moso bamboo plants. The primary hypotheses of this study were as follows: (1) N addition reduces foliar nutrient (N and P) resorption due to an increase in soil nutrient availability; (2) biochar amendment reduces foliar nutrient (N and P) resorption due to an increase in soil nutrient availability; and (3) the combination of biochar amendment and N addition decreases foliar nutrient (N and P) resorption to a greater degree than N addition alone.

## Materials and Methods

### Study Site

The experiment was conducted in the Lin’an District (30°14′ N, 119°42′ E), Hangzhou City, Zhejiang Province, China. The site has a subtropical monsoonal climate and distinct seasons. Mean annual air temperature and precipitation are 15.6°C and 1,420 mm, respectively. The topography consists of low hills, with an elevation of 100–300 m. The soil is classified as a Ferrosol and is derived from granite (Li and Feng, 2019).

Moso bamboo has a unique growth pattern. New bamboo shoots usually begin to emerge from the ground in March, and leaves appear in June of the same year. These leaves fall the following spring, and new leaves quickly emerge. These new leaves have a life span of 2 years and are, therefore, replaced biennially in spring (Zhang et al., 2017a). Moso bamboo forests are characterized by alternating high and low recruitment years. The recruitment of Moso bamboo shoots at the study site only occurred during even-numbered years (i.e., 2012, 2014, and 2016). Bamboo trunks are usually harvested after 4 years of growth to maximize economic benefits. Thus, Moso bamboo plantations are unevenly aged forests with leaves covering a two-year interval and usually have two growth stages (Song et al., 2016a). In our study site, Moso bamboo stands consisted of two growth stages: young bamboo shooting in the spring of 2016 (one-year-old) and mature bamboo shooting in the spring of 2014 (three-year-old). The initial stand and soil characteristics are listed in Supplementary Table A1 (Li et al., 2018b).

### Experimental Design

In November 2012, 12 plots of 20 × 20 m were established at the study site. Each plot was surrounded by a 20-m-wide buffer zone to avoid disturbing nearby plots. Based on the methods of Fang et al. (2007) and the local N deposition rate of 30 kg N ha^−1^ yr^−1^ (Jia et al., 2014), N was applied at a low (ambient + 30 kg N ha^−1^ yr^−1^, N30), medium (ambient + 60 kg N ha^−1^ yr^−1^, N60), and high (ambient + 90 kg N ha^−1^ yr^−1^, N90) rate, with control of ambient + 0 kg N ha^−1^ yr^−1^. These treatments were randomly applied to three replicate plots per treatment. According to the chemical composition (NH_4_^+^:NO_3_^−^ = 1.28) of wet N deposition in China, NH_4_NO_3_ of a similar composition ratio was selected as the simulated N source (Song et al., 2017b). Starting in January 2013, a quantitative NH_4_NO_3_ solution (10 L) was sprayed evenly on the forest floor of each plot at the beginning of each month, for 21 months. The same amount of water (N-free) was sprayed on each control plot in the same manner. In September 2014, following the 21-month period, we used a split-plot design to add biochar to each plot using three application rates (BC0, BC20, and BC40 representing 0, 20, and 40 t biochar ha^−1^, respectively) based on previous studies (Sun et al., 2014; Zhang et al., 2019a). Two subplots of 10 × 10 m and a subplot of 10 × 20 m were established within each plot (Supplementary Figure A1). Biochar was spread evenly over the ground and then thoroughly mixed into the top 30 cm of the soil by plowing. The remaining area inside the plot served as the control with no biochar application (BC0), which was plowed down to 30 cm. Sample plots were separated by aluminum-plastic plates. All subplots received monthly N additions, as described above. Biochar was produced by pyrolysis of Moso bamboo chips at 600°C (Yaoshi Coal Industry Co. Ltd., Hangzhou, China), and the particle size was less than 2 mm. The main characteristics of the biochar were pH (H_2_O) = 9.67; bulk density = 0.53 g cm^−3^; CEC = 14.9 cmol kg^−1^; carbon content = 81.73%; N content = 0.57%; and C:N ratio = 143.4.

### Leaf N and P Concentrations

We collected fresh leaf samples in July 2016 and senescent leaf samples in March 2017. Following the sampling methods of Song et al. (2020), three representative young Moso bamboo plants, with shoots emerging in April 2016, and three representative mature Moso bamboo plants, with shoots emerging in April 2014, were selected for sampling in each plot. According to previous studies (Zhang et al., 2019b; Li et al., 2021), 20 healthy leaves from the south-facing side of the mid-upper canopy were collected from the selected one-year-old and three-year-old plants. The leaves from the south-facing side were selected because these leaves have strong photosynthesis, good growth, and stable physiological structure (Zhang et al., 2017c). The leaf samples were transported to the laboratory in insulated cases at 4°C. Samples were dried at 105°C for 30 min and then dried at 65°C to a constant weight. Oven-dried leaves were milled for the analysis of N and P concentrations. Foliar N concentration was measured using an automatic CN analyzer (Sumigraph NC-80, Shimazu, Japan). Foliar P concentration was measured using the molybdenum antimony anti-colorimetric method (Lu, 2000).

Leaf nutrient RE (%) was calculated for NRE and PRE using the following equation:

$$\mathrm{RE}\;\left(\%\right)=\left(1-\mathrm{MLCF}*{\left[\mathrm{Nutrient}\right]}_{\mathrm{senescent}}/{\left[\mathrm{Nutrient}\right]}_{\mathrm{green}}\right)\times 100$$

where [Nutrient]_senescent_ and [Nutrient]_green_ are the concentrations of the nutrients in senesced leaves (March 2017) and green leaves (July 2016), respectively; MLCF is the mass loss correction factor that accounts for the mass loss during leaf senescence (Vergutz et al., 2012), calculated according to the ratio of the dry mass of 20 senesced leaves and 20 green leaves, with three replicates in each treatment (Supplementary Table A2); nutrient RP was quantified as the senescent leaf nutrient concentrations of N (NRP) and P (PRP; Killingbeck, 1996).

### Soil Nutrients

Six surface soil cores (0–20 cm) were collected randomly from each plot and mixed together to form a soil sample in March 2017. The samples were kept in a thermotank, transported to the laboratory, and then sieved through a 2-mm mesh to remove the roots, plant residue, and stones. The samples were air-dried and then used for the analysis of soil nutrient concentrations.

Soil pH was measured using a pH meter (FE20, Mettler Toledo, Switzerland) after shaking a soil–water (1:2.5 w/v) suspension for 30 min (Lu, 2000). Soil total N (TN) concentration was measured using an automatic CN analyzer (Sumigraph NC-80). Soil available N (AN) concentration was determined using the alkaline-KMnO_4_ method (Patrick and Wyatt, 1964). Soil total P (TP) concentration was determined by colorimetric analysis using a modified Kjeldahl method (Song et al., 2016a). Soil available P (AP) concentration was determined using the molybdenum blue method (Watanabe and Olsen, 1965).

### Statistical Analysis

Repeated-measures ANOVA was used to analyze the effects of interactions between N addition, biochar amendment, and bamboo age on foliar N and P concentrations, N:P ratios, NRE, PRE, and NRE:PRE ratios. One-way ANOVA and least significant difference (LSD) multiple comparisons were used to determine significant differences in foliar N and P concentrations, NRE, PRE, NRE:PRE ratios, and soil properties among treatments. All data were checked for normality and homogeneity of variance before testing for treatment differences. Principal components analysis (PCA) was used to identify the structure of the interdependencies between the soil properties and NRP, PRP, NRE, and PRE. Pearson’s correlation analysis was performed to test for correlations between leaf N and P concentrations, NRP, PRP, NRE, PRE, and soil properties. All statistical analyses in this study were conducted using the SPSS 22.0 software package for Windows (SPSS Inc., Chicago, IL, the United States).

## Results

### Soil Physical and Chemical Properties

Compared with the control, N addition alone significantly decreased soil pH and AN:AP ratios but significantly increased TN, TP, and AP concentrations (Supplementary Table A3). Soil pH, TN, TP, AN, and AP concentrations and AN:AP ratios were significantly higher in the biochar treatments than those in the control (Supplementary Table A3). Soil TN, AN, and AP concentrations and AN:AP ratios were significantly higher in the combined N (N30, N60, and N90) and biochar (BC20 and BC40) treatments than with N addition alone.

### Fresh Leaf N and P Concentrations

**Figure 1:**
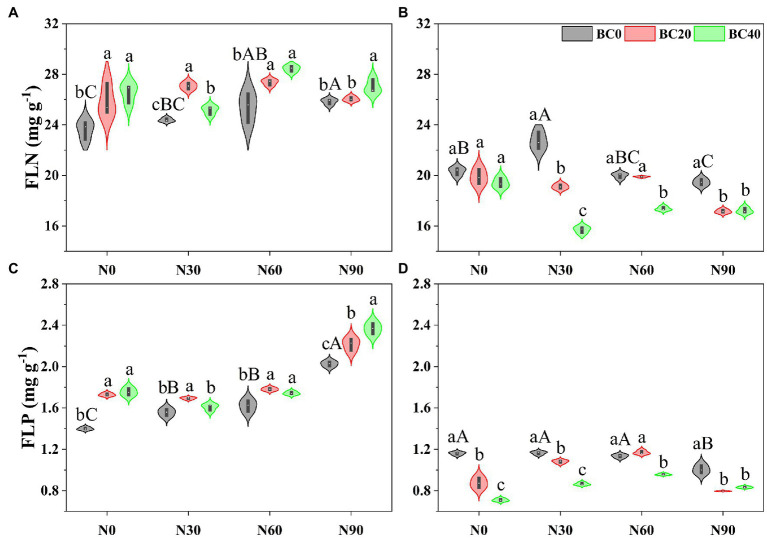
Fresh leaf nitrogen (FLN) and phosphorus (FLP) concentrations in young **(A,C)** and mature **(B,D)** bamboo under different N addition and biochar amendment treatments. N0, N30, N60, and N90 refers to 0, 30, 60, and 90 kg N ha^−1^ yr^−1^, respectively, and BC0, BC20, and BC40 refers to 0, 20, and 40 t biochar ha^−1^. Capital letters indicate a significant difference between different N addition treatments in the BC0 treatment at the 0.05 level. Lowercase letters indicate a significant difference between different biochar treatments in the same N addition treatment at the 0.05 level.

Repeated-measures ANOVA indicated that N addition, biochar amendment, and age of bamboo significantly affected fresh leaf N and P concentrations, both independently, and the two- and three-way interactions between variables (Table 1). In the control treatment, fresh leaf N and P concentrations in young bamboo were significantly higher than those in mature bamboo (Figure 1). Compared with the control, the N90 treatment significantly increased fresh leaf N and P concentrations in young bamboo but decreased these concentrations in mature bamboo (Figure 1). Biochar amendment alone significantly increased fresh leaf N (9.6–12.4%) and P (23.5–25.4%) concentrations in young bamboo (Figures 1A,C) and significantly decreased the fresh leaf P concentration in mature bamboo (24.6–38.8%, Figure 1D). In the N90 treatment, biochar amendment significantly increased fresh leaf N and P concentrations in young bamboo, while in mature bamboo, the opposite trend was observed (Figure 1).

**Table 1:** Repeated-measures ANOVA of the effects of N deposition (N), biochar amendment (BC), and age of bamboo on fresh leaf N (FLN) and P (FLP) concentrations, resorption proficiency, and resorption efficiency in the Moso bamboo plantation.

Difference source	Age		N		BC		Age*N		Age*BC		N*BC		Age*N*BC	
	F	P	F	P	F	P	F	P	F	P	F	P	F	P
FLN	3140.8	<0.001	10.541	<0.001	12.131	<0.001	29.15	<0.001	146.341	<0.001	21.678	<0.001	8.186	<0.001
FLP	11436.5	<0.001	235.302	<0.001	23.075	<0.001	520.904	<0.001	368.072	<0.001	20.878	<0.001	23.965	<0.001
NRP	6995.63	<0.001	394.331	<0.001	29.401	<0.001	76.188	<0.001	1075.7	<0.001	132.982	<0.001	50.868	<0.001
PRP	15,474	<0.001	966.837	<0.001	13.294	<0.001	545.389	<0.001	1505.91	<0.001	73.194	<0.001	53.324	<0.001
NRE	155.144	<0.001	465.271	<0.001	3.546	0.037	123.532	<0.001	452.849	<0.001	108.095	<0.001	56.543	<0.001
PRE	110.039	<0.001	254.142	<0.001	85.424	<0.001	91.866	<0.001	524.032	<0.001	79.369	<0.001	56.98	<0.001
NRE:PRE ratio	1.842	0.181	22.752	<0.001	44.356	<0.001	101.443	<0.001	9.197	<0.001	9.628	<0.001	12.516	<0.001

### Nutrient Resorption Proficiency

**Figure 2:**
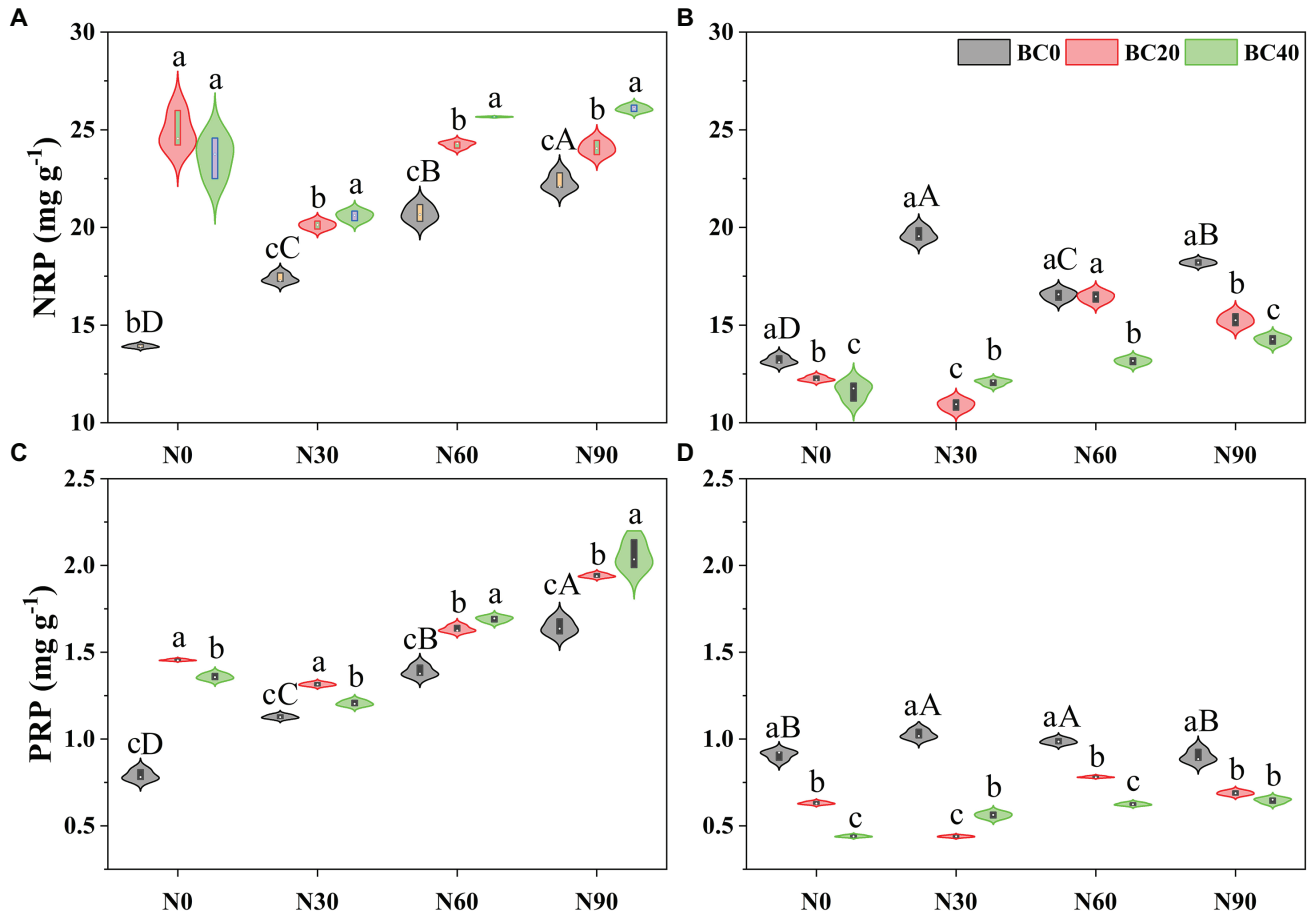
Nitrogen resorption proficiency (NRP) and phosphorus resorption proficiency (PRP) of young **(A,C)** and mature **(B,D)** bamboo under different N addition and biochar amendment treatments. N0, N30, N60, and N90 refer to 0, 30, 60, and 90 kg N ha^−1^ yr^−1^, respectively, and BC0, BC20, and BC40 refer to 0, 20, and 40 t biochar ha^−1^. Capital letters indicate a significant difference between different N addition treatments in the BC0 treatment at the 0.05 level. Lowercase letters indicate a significant difference between different biochar treatments in the same N addition treatment at the 0.05 level.

Repeated-measures ANOVA indicated that N addition, biochar amendment, and age of bamboo significantly affected the NRP and PRP, both independently, and the two- and three-way interactions between variables (Table 1). The NRP of young bamboo was higher than that of mature bamboo under the control treatment, while the PRP showed the opposite trend (Figure 2). Compared with the control, N addition alone significantly increased the NRP and PRP of young bamboo (25.0–60.2% and 42.7–108.1%, respectively) and mature bamboo (25.4–49.0% and 8.7–13.0%, respectively; Figures 2A,C). Compared with the control, biochar amendment alone significantly increased the NRP and PRP of young bamboo (69.5–79.0% and 71.9–84.1%, respectively; Figures 2A,C) but significantly decreased the NRP and PRP of mature bamboo (7.11–11.82% and 30.5–51.5%, respectively; Figures 2B,D). The foliar NRP and PRP of young bamboo were significantly higher in the combined N addition (N30, N60, and N90) and biochar amendment (BC20 and BC40) treatments than in the treatments with N addition only (Figures 2A,C), while those of mature bamboo showed the opposite trend (Figures 2B,D).

### Nutrient Resorption Efficiency

**Figure 3:**
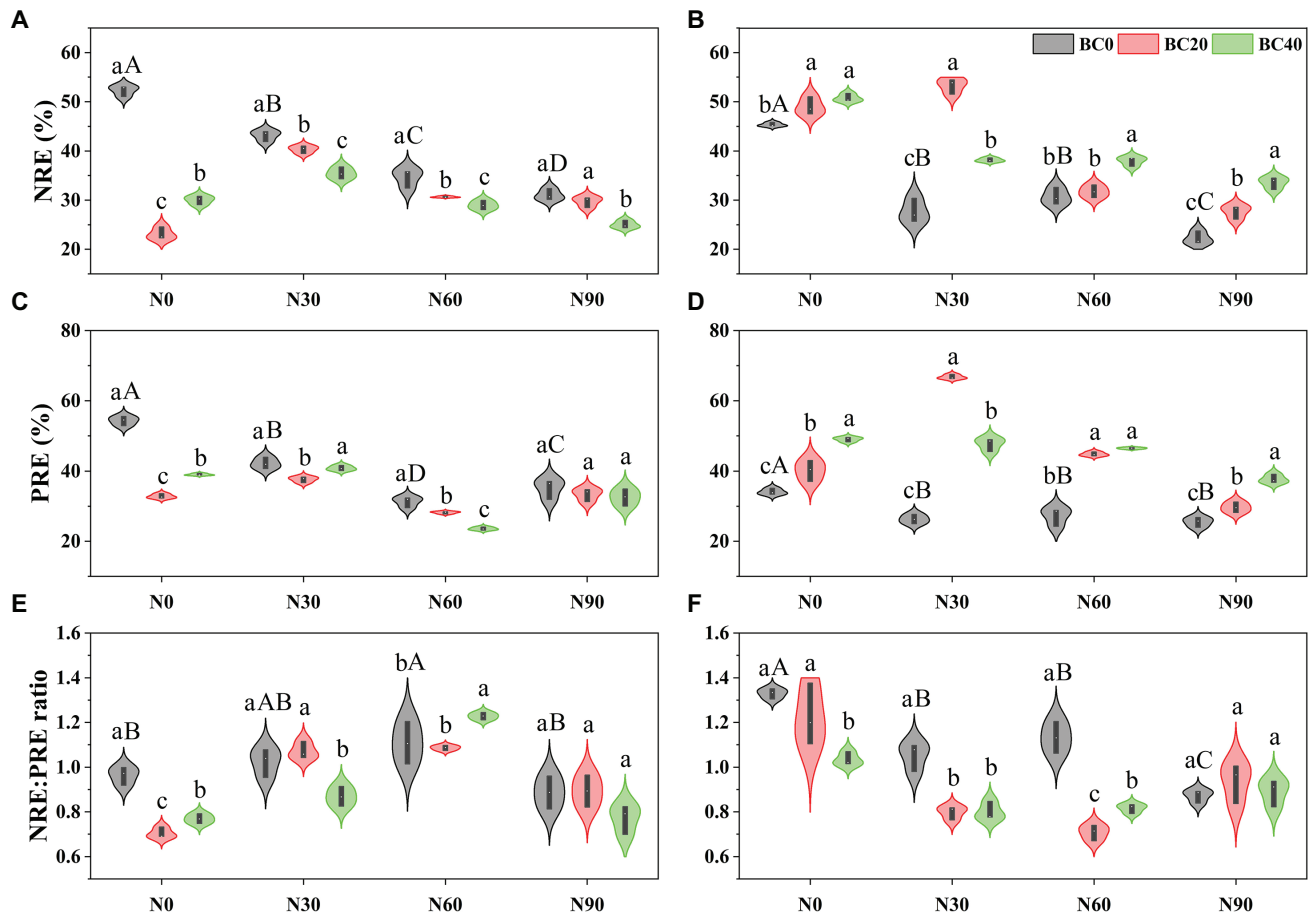
Nitrogen resorption efficiency (NRE), phosphorus resorption efficiency (PRE), and NRE:PRE ratio of young **(A,C,E)** and mature **(B,D,F)** bamboo under different N addition and biochar amendment treatments. N0, N30, N60, and N90 refer to 0, 30, 60, and 90 kg N ha^−1^ yr^−1^, respectively, and BC0, BC20, and BC40 refer to 0, 20, and 40 t biochar ha^−1^. Capital letters indicate a significant difference between different N addition treatments in the BC0 treatment at the 0.05 level. Lowercase letters indicate a significant difference between different biochar treatments in the same N addition treatment at the 0.05 level.

Repeated-measures ANOVA indicated that N addition, biochar amendment, and age of bamboo significantly affected NRE, PRE, and NRE:PRE ratios, both independently, and the two- and three-way interactions between variables (Table 1). Foliar NRE and PRE of young bamboo were significantly higher than those of mature bamboo in the control treatment, while the NRE:PRE ratio showed the opposite trend (Figure 3). Compared with the control, N addition alone significantly decreased the foliar NRE and PRE of young (17.5–40.6% and 22.3–42.4%, respectively) and mature bamboo (32.3–51.0% and 20.2–25.3%, respectively; Figure 3). Biochar amendment alone significantly decreased the NRE and PRE of young bamboo (42.6–55.8% and 28.2–39.6%, respectively) but significantly increased those of mature bamboo (8.1–12.0% and 17.8–43.8%, respectively) relative to that of the control. Foliar NRE and PRE of young bamboo were significantly lower in the combined N addition (N30, N60, and N90) and biochar amendment (BC20 and BC40) treatments than those in the N-addition treatments alone (Figures 3A,C), whereas mature bamboo showed the opposite trend (Figures 3B,D). N addition (N30 and N60) significantly increased the NRE:PRE ratio of young bamboo (6.3–15.1%, Figure 3E) relative to that of the control, but significantly decreased that of mature bamboo (14.8–34.4%, Figure 3F); biochar amendment significantly decreased the NRE:PRE ratio of young (20.2–26.9%) and mature bamboo (7.8–22.2%, Figures 3E,F). In the N30 treatment, BC40 significantly decreased the NRE:PRE ratio of young bamboo by 15.2% (Figure 3E), while in the N60 treatment, BC40 significantly increased the NRE:PRE ratio of young bamboo by 10.8% (Figure 3E). The foliar NRE:PRE ratio of mature bamboo was significantly lower in the combined N addition (N30 and N60) and biochar amendment (BC20 and BC40) treatments than that in the N-addition treatments alone (Figure 3F).

### Relationship Between Soil Properties, Nutrient Resorption Proficiency, and Nutrient Resorption Efficiency

**Figure 4:**
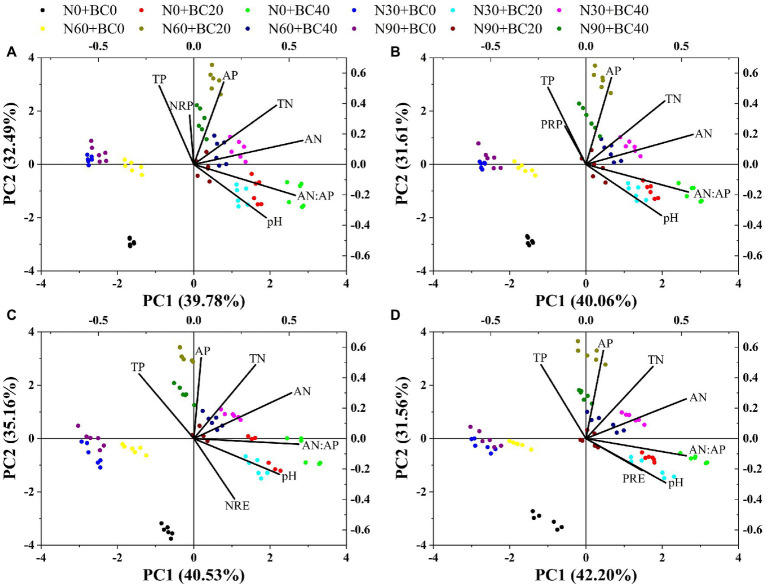
Principal components analysis (PCA) of NRP **(A)**, PRP **(B)**, NRE **(C)**, and PRE **(D)**, and soil properties. TN, soil total nitrogen; TP, soil total phosphorus; AN, soil available nitrogen; AP, soil available phosphorus.

The PCA showed that the first two principal components could explain 72.27% (Figure 4A), 71.67% (Figure 4B), 75.69% (Figure 4C), and 73.76% (Figure 4D) of the variance in NRP, PRP, NRE, and PRE, respectively. For NRP, PRP, NRE, and PRE, the control treatments were found in the lower-left corner of the plot and were separated from the N addition and biochar amendment treatments (Figure 4). Soil pH, soil TN, TP, AN, and AP concentrations, and AN:AP ratio showed similar interactions and much greater interaction than NRP, PRP, NRE, and PRE.

NRP was significantly positively correlated with fresh N and P concentrations and soil TP concentration but was significantly negatively correlated with soil pH (*p* < 0.05, Table 2). PRP was significantly positively correlated with fresh N and P concentrations, but significantly negatively correlated with soil pH (*p* < 0.01, Table 2). NRE was significantly negatively correlated with fresh N and P concentrations, soil TN, TP, and AP concentrations, but significantly positively correlated with soil pH (*p* < 0.05, Table 2). PRE was significantly negatively correlated with fresh N and P concentrations and soil TP concentration, but significantly positively correlated with AN, pH, and AN:AP ratio (*p* < 0.05, Table 2).

**Table 2:** Pearson’s correlation coefficients indicating the relationships between N and P resorption proficiency (NRP and PRP), N and P resorption efficiency (NRE and PRE), and NRE:PRE ratio and fresh leaf N and P concentrations and soil characteristics in a Moso bamboo plantation.

Difference source	FLN	FLP	pH	TN	TP	AN	AP	AN:AP ratio
NRP	0.864^∗∗^	0.866^∗∗^	−0.244^∗^	0.201	0.274^∗^	0.038	0.165	−0.059
PRP	0.870^∗∗^	0.949^∗∗^	−0.283^∗^	0.076	0.175	−0.049	0.132	−0.139
NRE	−0.275^∗^	−0.384^∗∗^	0.482^∗∗^	−0.263^∗^	−0.457^∗∗^	0.035	−0.275^∗^	0.224
PRE	−0.354^∗∗^	−0.293^∗^	0.462^∗∗^	−0.006	−0.296^∗^	0.284^∗^	−0.021	0.316^∗∗^

FLN, fresh leaf N concentration; FLP, fresh leaf P concentration; TN, soil total nitrogen; TP, soil total phosphorus; AN, soil available nitrogen; AP, soil available phosphorus.

∗ *p* < 0.05;

∗∗ *p* < 0.01.

## Discussion

### Effect of N Addition on Foliar Nutrient Resorption

In the present study, foliar NRE (45–52%) and PRE (34–54%) of Moso bamboo in the control plots were lower than the global average NRE and PRE of evergreen angiosperms (56 and 58%, respectively; Vergutz et al., 2012), which may be attributed to the different species and soil nutrients. Previous studies demonstrated that tree species with a high RE and low RP adopt a “conservative consumption” nutrient-use strategy, whereas those with a low RE and high RP adopt a “resource spending” strategy (Wright and Westoby, 2003; Wu et al., 2020). In the present study, the foliar NRE and PRE of young bamboo in the control treatment were higher than those of the mature bamboo, whereas foliar PRP exhibited the opposite trend. These results were similar to the findings of Wu et al. (2020), who found that leaf NRE and PRE decreased with stand age in Chinese fir (*Cunninghamia lanceolata*) plantations, indicating that young bamboo adopts a “conservative consumption” strategy, while mature bamboo adopts a “resource spending” strategy. A possible explanation for this strategy is that rapid growth in young bamboo increases nutrient demands and further promotes plant nutrient uptake from the soil and nutrient resorption, according to the growth rate hypothesis (Delgado-Baquerizo et al., 2016; Achat et al., 2018; Wu et al., 2020; Li et al., 2021). In addition, our previous studies at the same site demonstrated that young bamboo had significantly greater foliar N and P concentrations and N:P ratios but lower NRE:PRE ratios than those of mature bamboo (Song et al., 2016a; Li et al., 2021), which directly supported the point that young and mature bamboo had different nutrient absorption strategy. Plant leaf NRE and PRE are used to estimate N and P limitations at the ecosystem scale according to the stoichiometric homeostasis theory and Liebig’s law of the minimum (Hooker, 1917; Du et al., 2020). A leaf NRE:PRE ratio >1 indicates a stronger N limitation than P limitation. Alternatively, P may be more limiting when the leaf NRE:PRE ratio is <1 (Du et al., 2020). In this study, the foliar NRE:PRE ratio of young bamboo (<1) in the control treatment was significantly different from that of mature bamboo (>1), indicating that young bamboo experienced P limitation, while mature bamboo experienced N limitation.

In the present study, N addition significantly reduced the foliar NRE and PRE but increased the NRP and PRP of young and mature bamboo, which supports our first hypothesis and indicates that N addition alters the nutrient-use strategy of Moso bamboo from “conservative consumption” to “resource spending.” Previous studies have reported that N addition decreased plant N resorption (Lü et al., 2013; Yuan and Chen, 2015; Zhao et al., 2020). For example, Zhang et al. (2017b) found that N input significantly decreased the leaf NRE of Chinese fir. In our study, N addition significantly increased soil TN concentration and decreased foliar NRE. A review by Yuan and Chen (2015) demonstrated that plant nutrient resorption decreased with increasing soil nutrient availability. Pearson’s correlation analysis showed that foliar NRE was significantly negatively correlated with soil TN concentration, which supports our argument that high nutrient availability reduces plant nutrient resorption. Moreover, enhanced N availability affected the resorption of other elements (Brant and Chen, 2015; See et al., 2015). The effect of N addition on foliar PRE is highly variable, with negative, neutral, and positive effects reported (van Heerwaarden et al., 2003; Lü et al., 2013, 2020; Kou et al., 2017). Kou et al. (2017) observed that N enrichment led to increased P resorption, as plants increased P conservation during the transition from N limitation to P limitation. However, some studies found that N enrichment lowered P resorption (van Heerwaarden et al., 2003; Lü et al., 2013, 2020) because N enrichment enhanced soil P availability by stimulating extracellular phosphatase enzyme activity (Chen et al., 2020; Lü et al., 2020). In the present study, and in previous studies at the same site, N addition significantly increased soil TP and AP concentrations (Supplementary Table A3) and acid phosphatase enzyme activity (Peng et al., 2019) and then decreased PRE. In addition, foliar PRE was significantly negatively correlated with soil TP concentration (Supplementary Table A3), which supports the argument that N addition decreases foliar PRE by increasing soil P availability. Additionally, some studies have demonstrated a negative correlation between N and P resorption efficiency and fresh leaf N and P concentrations (Kobe et al., 2005; Vergutz et al., 2012). Our study found that fresh leaf N and P concentrations were significantly higher in the N-addition treatments (Figure 1) and were negatively correlated with foliar NRE and PRE, which supports the above argument that N addition decreases nutritional resorption by increasing foliar nutrient concentrations.

Previous studies have suggested that nutrient RP is more sensitive than RE to nutrient addition (Ratnam et al., 2008; Yuan and Chen, 2015) and is less variable over time (Killingbeck, 1996; Lü et al., 2020). These findings have been attributed to RE being calculated from percent changes in the nutrient content of green and senesced leaves. In this study, N addition significantly increased the NRP and PRP of young and mature bamboo, which is consistent with the findings of Yuan and Chen (2015) and Lü et al. (2020). A possible reason for this result is that N addition increased soil nutrient availability (TN and TP, Supplementary Table A3).

Thus, our study showed that young and mature Moso bamboo had a higher RE and lower RP in the N-addition treatments, indicating that N addition altered the nutrient-use strategy of Moso bamboo from “conservative consumption” to “resource spending.” In addition, the NRE:PRE ratio of mature bamboo was significantly lower in the N-addition treatments than that in the N-free treatment, which did not exceed 1. The result indicates that proportionally more P than N was resorbed, suggesting that N addition changed the nutrition limitation of mature bamboo from N limitation to P limitation.

### Effect of Biochar Amendment on Foliar Nutrient Resorption

Biochar amendment significantly reduced the NRE and PRE but increased the NRP and PRP of young bamboo, supporting our second hypothesis. Biochar contains abundant nutrients (e.g., NH_4_^+^, ortho-P; Gul and Whalen, 2016); its application in the experimental plots increased the soil AN and AP content (Supplementary Table A3), thus reducing the plants’ dependence on internal circulation of N and P, leading to decreased N and P resorption in young bamboo. Zhang et al. (2017d) also observed that biochar amendment increased soil nutrient availability in *Torreya grandis* plantations. Moreover, biochar application promoted the formation of soil aggregates (Brodowski et al., 2006) and greatly enhanced soil fertility, which is attributed to biochar having a highly porous structure, large specific surface area, and a high CEC (Bird et al., 2008; Cheng et al., 2008).

In the present study, NRE and PRE were significantly and negatively correlated with soil TN, TP, and AP concentrations (Supplementary Table A3), supporting our hypothesis that biochar amendment reduces NRE and PRE by increasing soil nutrient concentrations. In addition, green leaf N and P concentrations in young bamboo were significantly higher in the BC20 and BC40 treatments than those in the control (Figure 1) and were significantly and negatively correlated with the foliar NRE and PRE of young bamboo. These results support the argument that high green leaf N and P concentrations decrease nutrient RE. However, biochar amendment significantly decreased green leaf N and P concentrations in mature bamboo (Figure 1) but increased foliar N and P resorption. Thus, the different responses of young and mature bamboo to biochar amendment may depend on the growth stage of the plant. These findings suggest that young bamboo adopts a “resource spending” strategy (with low RE and high RP), whereas mature bamboo adopts a “conservative consumption” strategy (with high RE and low RP) in response to biochar amendment. Biochar application significantly decreased the foliar NRE:PRE ratios of young (<1) and mature bamboo (>1), indicating enhanced P limitation in young bamboo and alleviated N limitation in mature bamboo.

### Combined Effects of N Addition and Biochar Amendment

The combined application of biochar and N significantly decreased foliar NRE and PRE but increased NRP and PRP of young bamboo when compared with those with N addition alone. These findings support our third hypothesis and demonstrate that biochar amendment enhanced the negative priming effect of N addition on foliar nutrient resorption in young Moso bamboo. A possible explanation could be that the combined application of biochar and N significantly increased soil N and P availability (Supplementary Table A3), thereby increasing the fresh leaf N and P concentrations in young bamboo. The combined applications of biochar and N increased soil AP and TP concentrations (Asai et al., 2019), which significantly enhanced fresh leaf N and P concentrations in *T. grandis* trees and seedlings (Zhang et al., 2019b) and wheat (Huong, 2016). However, mature bamboo showed the opposite response to combined biochar and N applications, suggesting that biochar amendment alleviates the negative priming effect of N addition on foliar nutrient resorption in mature bamboo. In the N30 and N60 treatments, biochar application significantly decreased the foliar NRE:PRE ratio of mature bamboo (<1) relative to that in the N-addition-alone treatments, indicating that biochar amendment enhanced P limitation in mature bamboo. However, the foliar NRE:PRE ratios of young bamboo in the combined N60 and N90 and BC20 and BC40 treatments were similar to those of the N-addition treatments alone (both greater than or less than 1), suggesting that biochar amendments did not change the effect of N addition on nutrient limitations in young bamboo.

## Conclusion

Our findings show that N addition alone significantly decreased leaf nutrient resorption (NRE and PRE) in both young and mature bamboo, indicating that N deposition reduced internal nutrient cycling and altered the nutrient-use strategy of Moso bamboo from “conservative consumption” to “resource spending.” Biochar amendment alone significantly decreased the leaf NRE and PRE of young bamboo but increased that of mature bamboo, suggesting that the effect of biochar amendment on nutrition resorption depends on the age of the bamboo. Furthermore, biochar combined with N significantly decreased the leaf NRE and PRE of young bamboo but increased that of mature bamboo, indicating that biochar amendment altered the negative priming effect of N addition on leaf nutrition resorption, and the effect direction depended on bamboo age. There was a negative correlation between NRE and PRE and fresh leaf N and P concentrations and soil nutrient content. These findings will assist policymakers and managers with establishing efficient fertilization management strategies under changing N deposition rates in subtropical Moso bamboo plantations. Besides, the long-term effect of biochar amendment on Moso bamboo nutrient resorption should be evaluated in future studies.

## Data Availability Statement

The original contributions presented in the study are included in the article/Supplementary Material, further inquiries can be directed to the corresponding author.

## Author Contributions

XS designed the research. JG and QL performed the research and collected and analyzed data. All authors contributed to the article and approved the submitted version.

### Conflict of Interest

The authors declare that the research was conducted in the absence of any commercial or financial relationships that could be construed as a potential conflict of interest.
