# Supplementary material for: Biochar Amendment Alters the Nutrient-Use Strategy of Moso Bamboo Under N Additions
Source: Front Plant Sci. 2021 Jun 23;12:667964. doi: 10.3389/fpls.2021.667964 (PMC8261046; doi:10.3389/fpls.2021.667964)

**Table A1.** Original stand and surface soil (0–20 cm) characteristics of study sites in the Moso bamboo forest (Mean ± SD, n=3).

| Nitrogen treatment | SD (trees·hm^-2^) | DBH (cm) | SBD (g·cm^-3^) | SOC (mg·g^-1^) | TN (mg·g^-1^) | TP (mg·g^-1^) | pH |
| --- | --- | --- | --- | --- | --- | --- | --- |
| Control | 3362±309 | 10.16±0.13 | 0.97±0.07 | 23.73±0.24 | 1.11±0.04 | 0.52±0.01 | 4.46±0.01 |
| N30 | 3408±382 | 10.31±0.32 | 0.98±0.04 | 22.56±1.17 | 1.17±0.09 | 0.57±0.05 | 4.43±0.04 |
| N60 | 3208±187 | 10.76±0.59 | 1.00±0.05 | 23.15±1.74 | 1.21±0.12 | 0.54±0.03 | 4.48±0.08 |
| N90 | 3250±100 | 10.66±0.48 | 0.94±0.03 | 25.34±2.16 | 1.13±0.06 | 0.56±0.03 | 4.42±0.06 |

SD: stand density; DBH: diameter at breast height; SD: soil bulk density; SOC: soil organic carbon; TN: soil total nitrogen; TP: soil total phosphorus

**Table A2.** Mass loss correction factor of Moso bamboo forest.

| Treatment | Young bamboo | Mature bamboo |
| --- | --- | --- |
| N0+BC0 | 0.809±0.005aA | 0.842±0.003aA |
| N0+BC20 | 0.798±0.003b | 0.827±0.002b |
| N0+BC40 | 0.787±0.005c | 0.821±0.005b |
| N30+BC0 | 0.797±0.008abB | 0.837±0.004aAB |
| N30+BC20 | 0.803±0.002a | 0.818±0.002b |
| N30+BC40 | 0.788±0.001b | 0.804±0.003c |
| N60+BC0 | 0.801±0.004aAB | 0.837±0.005aAB |
| N60+BC20 | 0.783±0.004b | 0.825±0.002b |
| N60+BC40 | 0.787±0.003b | 0.819±0.001b |
| N90+BC0 | 0.799±0.004aAB | 0.831±0.003aB |
| N90+BC20 | 0.760±0.001c | 0.813±0.002b |
| N90+BC40 | 0.776±0.005b | 0.803±0.001c |

Capital letters indicate a significant difference between different N addition treatments in the BC0 treatment at the 0.05 level. Lowercase letters indicate a significant difference between different biochar treatments in the same N addition treatment at the 0.05 level.

**Table A3.** Soil physical and chemical properties in experimental plots in a Moso bamboo forest treated with different N (N0, N30, N60, and N90 is 0, 30, 60, and 90 kg N ha^-1^ yr^-1^, respectively) and biochar (BC0, BC20, and BC40 is 0, 20, and 40 t biochar ha^-1^) applications.

| Treatment | pH | TN (g·kg^-1^) | TP (g·kg^-1^) | AN (mg·kg^-1^) | AP (mg·kg^-1^) | AN:AP ratio |
| --- | --- | --- | --- | --- | --- | --- |
| N0+BC0 | 4.667±0.031bA | 1.273±0.052cD | 0.412±0.006bD | 57.400±2.090cB | 13.545±0.243cC | 4.240±0.230cA |
| N0+BC20 | 4.750±0.020a | 1.998±0.049b | 0.490±0.004a | 202.113±4.277b | 19.304±0.751a | 10.478±0.379b |
| N0+BC40 | 4.773±0.025a | 2.281±0.052a | 0.487±0.001a | 219.847±14.023a | 16.004±0.115b | 13.736±0.853a |
| N30+BC0 | 4.443±0.031cB | 1.526±0.031cC | 0.653±0.005aA | 40.527±1.901bD | 18.368±0.785cA | 2.209±0.129cC |
| N30+BC20 | 4.577±0.050b | 1.919±0.014b | 0.439±0.009c | 203.980±4.850a | 20.354±0.257b | 10.021±0.171a |
| N30+BC40 | 4.660±0.020a | 2.128±0.062a | 0.541±0.033b | 207.247±0.808a | 31.838±0.577a | 6.511±0.113b |
| N60+BC0 | 4.297±0.057bC | 1.978±0.034cA | 0.473±0.016cC | 65.806±0.562cA | 18.941±0.373cA | 3.475±0.049cB |
| N60+BC20 | 4.393±0.042ab | 2.378±0.043a | 0.662±0.033a | 206.313±3.233a | 40.788±0.656a | 5.058±0.052b |
| N60+BC40 | 4.463±0.051a | 2.149±0.018b | 0.540±0.003b | 186.713±2.139b | 22.504±1.097b | 8.307±0.326a |
| N90+BC0 | 4.303±0.065bC | 1.736±0.037bB | 0.610±0.023aB | 50.376±1.802cC | 16.203±1.283cB | 3.126±0.324cB |
| N90+BC20 | 4.383±0.025ab | 1.968±0.068a | 0.492±0.025c | 174.113±3.233b | 19.671±0.501b | 8.858±0.383a |
| N90+BC40 | 4.473±0.065a | 2.041±0.069a | 0.550±0.008b | 181.113±3.523a | 38.438±0.150a | 4.712±0.103b |

TN, soil total nitrogen; TP, soil total phosphorus; AN, soil available nitrogen; AP, soil available phosphorus. Capital letters indicate a significant difference between different N addition treatments in the BC0 treatment at the 0.05 level. Lowercase letters indicate a significant difference between different biochar treatments in the same N addition treatment at the 0.05 level.

**Figure caption**

**Fig. A1. Layout of experiment site.**

**Figure**

Fig. A1


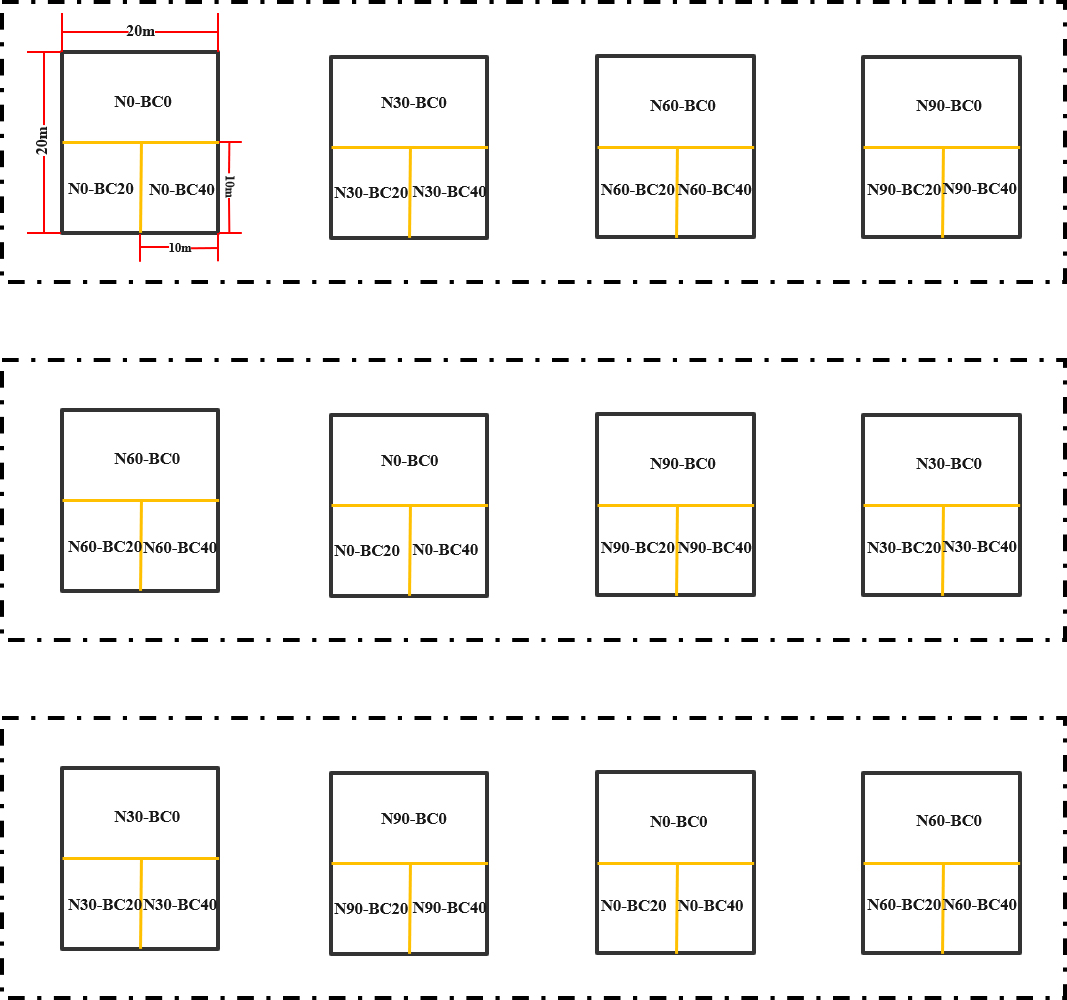

Supplement: Supplementary file 1 [file Data_Sheet_1.docx]
